# Risks of Polymyxin B Nephrotoxicity and Its Precursors in the Intensive Care Unit: A Retrospective Cohort Study

**DOI:** 10.7759/cureus.44301

**Published:** 2023-08-29

**Authors:** Hüseyin Özkarakaş, Yeliz Özdemir, Selma Tosun, Zeki T Tekgül, Mehmet U Bilgin, Ozkan Özmuk, Bülent Çalık

**Affiliations:** 1 Intensive Care Unit, University of Health Sciences Izmir Bozyaka Training and Research Hospital, Izmir, TUR; 2 Infectious Diseases and Clinical Microbiology, University of Health Sciences Izmir Bozyaka Training and Research Hospital, Izmir, TUR; 3 Anesthesiology and Reanimation, University of Health Sciences Izmir Bozyaka Training and Research Hospital, Izmir, TUR; 4 Anesthesiology and Reanimation, Helios Klinikum Schleswig, Academic Teaching Hospital for the University of Kiel and Lubeck, Schleswig, DEU; 5 Critical Care Medicine, University of Health Sciences Izmir Bozyaka Training and Research Hospital, Izmir, TUR; 6 General Surgery, University of Health Sciences Izmir Bozyaka Training and Research Hospital, Izmir, TUR

**Keywords:** carbapenem resistant gram negative infection, sepsis, nephrotoxicity, i̇ntensive care unit, acute kidney injury, polymyxin-b

## Abstract

Background and Aim: Polymyxin group antibiotics constitute a part of our limited arsenal in the treatment of multidrug-resistant gram-negative bacteria. However, their use is limited especially due to nephrotoxicity and other side effects. In this study, we primarily aimed to determine the effect of polymyxin B on the rate of nephrotoxicity in critically ill patients, and secondly to identify the factors that facilitate nephrotoxicity caused by polymyxin B.

Materials and Methods: The study was designed as a retrospective cohort study and conducted by scanning patients aged 18 years or older who had been admitted to our intensive care unit (ICU) in 2022 and treated with polymyxin B for at least 72 hours. Patients without chronic renal failure and acute kidney injury (AKI) before starting polymyxin B therapy were included and AKI was examined after the use of polymyxin B. The patients were then divided into two groups, those with AKI and those without AKI. We tried to find factors that may facilitate AKI by comparing the two groups.

Results: Of the patients, 26 were female and 34 were male. In 21 of the patients (35%), renal damage of varying degrees developed; these patients belonged to the nephrotoxicity (NT) group, while the rest belonged to the non-nephrotoxicity (non-NT) group. We found that advanced age (p=0.008), low baseline GFR (p=0.01), baseline creatinine (p=0.006), BMI (p=0.011), concomitant diseases (p<0.001), and days of use of polymyxin B (p=0.006) were statistically different between the two groups. In multivariate analysis of univariate analysis, we found that duration of polymyxin B use, BMI, and advanced age were independent risk factors for AKI development.

Conclusion: We found that 21 (35%) of 60 intensive care unit patients who had no previous history of kidney injury developed kidney injury after being treated with polymyxin B. We identified advanced age, high BMI, and duration of polymyxin B use as independent risk factors. Therefore, we recommend close monitoring of renal function and prompt intervention, particularly in patients with risk factors, during polymyxin B use.

## Introduction

The polymyxin group of antibiotics was synthesized in the 1950s and used to treat gram-negative bacteria. However, due to many side effects and the introduction of new antibiotics, their use gradually declined [1,2]. In the past two to three decades, however, older, out-of-use antibiotics have regained importance due to the increasing prevalence of multidrug-resistant gram-negative bacteria and unsuccessful attempts at the synthesis of new antibiotics to combat these bacteria. The polymyxin group constitutes a small part of our limited arsenal against multidrug-resistant bacteria. However, the risk of nephrotoxicity and neurotoxicity severely constrain the use of these drugs. Kidney damage is especially significant in critically ill patients, as such damage places an additional burden on the patient already suffering from existing diseases and the drugs used to treat them in intensive care, which can facilitate the development of renal damage.

Various studies [3,4] indicate the incidence of colistin nephrotoxicity to be between 14% and 76%. Nephrotoxicity caused by polymyxin B is less common than colistin nephrotoxicity, but the range is nonetheless broad (4%-60%) [5,6]. Studies suggest that the main factors increasing nephrotoxicity are the loading dose and the use of additional nephrotoxic agents.

Intravenous use of polymyxin B is relatively new in our country. The drug was licensed in 2018 and deployed in 2020. Naturally, numerous clinical studies can be found on colistin, while the number of clinical studies on polymyxin B is much fewer.

## Materials and methods

Our study was planned as a single-center retrospective cohort study and conducted in the second- and third-level intensive care units (ICUs) of the University of Health Sciences Izmir Bozyaka Training and Research Hospital. Patients who received polymyxin B for at least 72 hours in 2022 were included in our study. The ethical committee of our hospital reviewed the study and approved it on December 12, 2022, with the approval number 363.

The primary objective of this study is to determine the effect of polymyxin B on the rate of nephrotoxicity in critically ill patients, and our secondary objective is to identify the factors that facilitate nephrotoxicity caused by polymyxin B.

**Figure 1:**
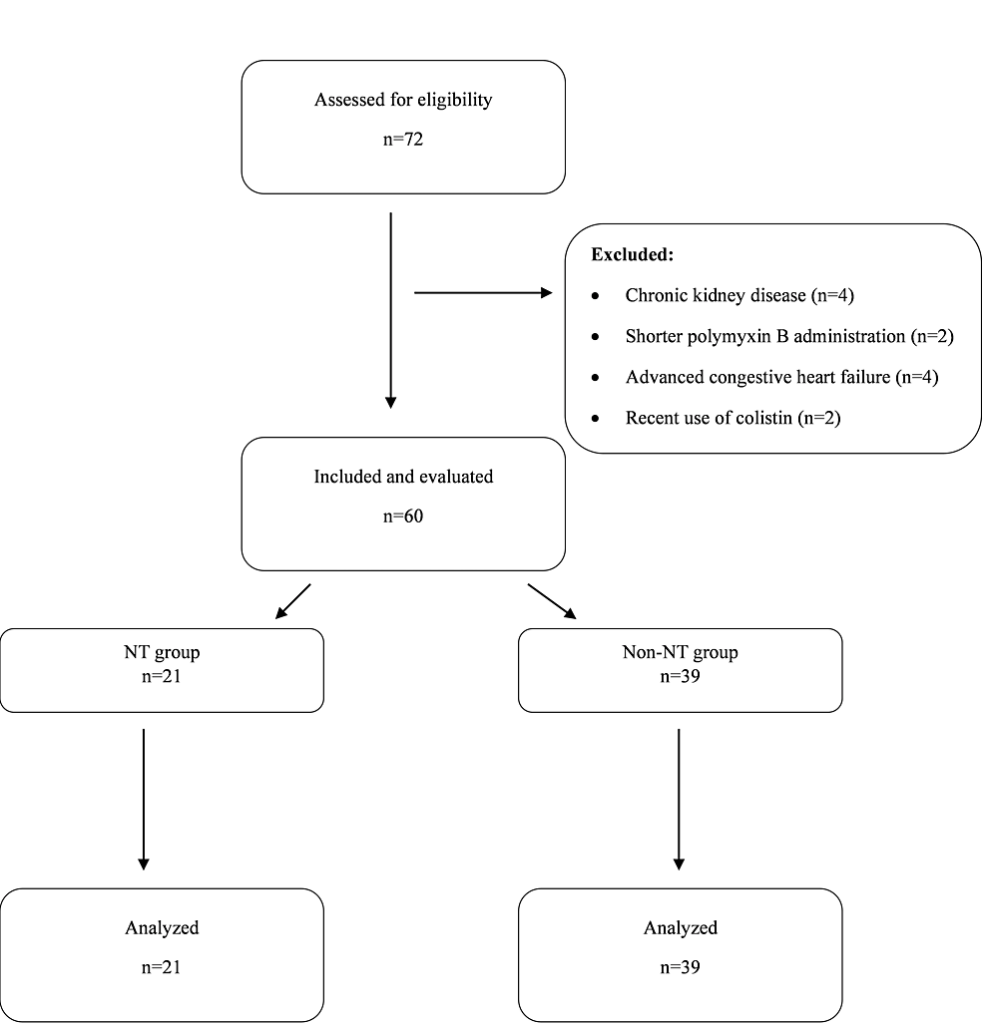
Flow chart NT: nephrotoxicity

Patients without a previous history of chronic renal failure or acute kidney injury (AKI), aged 18 years or over who had a hospital-acquired multidrug-resistant gram-negative infection (e.g., *Acinetobacter baumannii*, *Klebsiella pneumonia*, *Pseudomonas aeruginosa*, or other enterobacteriaceae) and who were started on intravenous polymyxin B therapy by an infectious disease specialist were included in our study. Patients who received polymyxin B for less than 72 hours, had chronic kidney failure, underwent active hemodialysis, had a cancer diagnosis, had severe heart failure (EF<20%), received intravenous colistin up to 15 days before the start of polymyxin therapy, or were pregnant were excluded from the study (Figure 1). All patients received 1.5 mg/kg of polymyxin B (Polix 500,000 IU, Koçak Farma), divided into two doses per day, following a loading dose of 2.5 mg/kg.

Data for our study were obtained through the electronic patient record system of the hospital between January 1 and December 31, 2022. Patients’ age, gender, baseline creatinine, baseline glomerular filtration rate (GFR) (calculated according to the short Modification of Diet in Renal Disease “MDRD” formula), Acute Physiology and Chronic Health Evaluation-2 (APACHE-2) score, serum albumin value before polymyxin B therapy, hemoglobin, lactate value, type and location of infection that led to polymyxin B use, the use of other nephrotoxic agents (e.g., angiotensin-converting-enzyme inhibitors, nonsteroidal anti-inflammatory drugs, or other nephrotoxic antibiotics), and vasopressor agent use were recorded. The urea and creatinine levels and GFR values of the patients were followed from the start of polymyxin B use to seven days after its end, and the highest value was evaluated. Renal damage was classified according to the Kidney Disease Improving Global Outcomes (KDIGO) [7] guidelines (Table 1) between stages 1 and 3. Urine output, in accordance with the KDIGO guidelines, was not evaluated due to difficulty in reaching the patients’ exact urine output and data security.

**Table 1:** KDIGO definition of acute kidney injury [7] eGFR: estimated glomerular filtration rate, KDIGO: Kidney Disease Improving Global Outcomes

Stage	Serum creatinine	Urine output
Stage 1	1.5-1.9 times baseline OR ≥0.3 mg/dl (≥26.5 mmol/l) increase	<0.5 ml/kg/h for 6-12 hours
Stage 2	2.0-2.9 times baseline	<0.5 ml/kg/h for ≥12 hours
Stage 3	3.0 times baseline OR increase in serum creatinine to ≥4.0 mg/dl (≥353.6 mmol/l) OR initiation of renal replacement therapy OR in patients <18 years, decrease in eGFR to ≤35 ml/min per 1.73 m2	<0.3 ml/kg/h for ≥24 hours OR anuria for ≥12 hours

Statistical analysis

IBM SPSS version 22.0 (IBM Corp, Armonk, NY, USA) was used for statistical analysis. Normality was assessed using the Kolmogorov-Smirnov test. Continuous normally distributed data, expressed as mean ± SD, were compared using an independent sample t-test. Non-normally distributed data, expressed as the median and interquartile range (IQR), were compared using the Mann-Whitney U test. Categorical data are expressed as a number (n) and percentage (%) of events and compared by Pearson’s chi-squared or Fisher’s exact test. The data were analyzed at a 95% confidence level. A p-value of less than 0.05 was considered statistically significant.

The receiver operating characteristic (ROC) curve was used to evaluate the cut-off values of independent numerical variables with a p-value less than 0.05. The Youden’s indices were calculated, and the maximum Youden’s index was used as the cut-off value in the ROC curve. Cut-off values for nephrotoxicity and mortality were separately analyzed. Area under the curve (AUC) values of 0.9-0.99, 0.8-0.

## Results

It was observed that polymyxin B was used in a total of 72 patients between January 1 and December 31, 2022. Twelve of these patients were excluded from the study due to various reasons. Sixty patients in total were evaluated (Figure 1). Of the patients, 26 were female and 34 were male. The demographic and clinical information of the patients is presented in Table 2.

**Table 2:** Demographic data and first-day laboratory data a: first day, b: number and %, * median (interquartile range), c: chi-squared test, d: Mann-Whitney U test, f: Student’s t-test, NT: nephrotoxicity, APACHE: Acute Physiology and Chronic Health Evaluation, AST: aspartate aminotransferase, ALT: alanine aminotransferase, GFR: glomerular filtration rate

	NT group	Non-NT group	p-value
Gender^c^			0.548
Female^b^	8 (38.1%)	18 (46.2%)	
Male^b^	13 (61.9%)	21 (53.8%)	
Height*^,d^	165 (11.5)	165 (10)	0.241
Weight*^,d^	75 (10)	65 (11)	<0.001
BMI*^,d^ (kg/m2)	25.5 (4)	23.14 (3)	<0.001
APACHE*^,d^	14 (17)	21 (16)	0.055
Age*^,f^	72 (17)	52 (46)	0.008
Urea*^,d^ (mg/dl)	109 (68)	58 (72)	0.024
Creatinine*^,a,d^ (mg/dl)	1.3 (0.8)	0.6 (0.9)	0.006
GFR*^,a,d^	57.23 (39.82)	139.23 (168.62)	0.01
Procalsitonin*^,a,d ^(mg/dl)	1.3 (8.15)	2.5 (3.5)	0.914
AST*^,a,d ^(U/L)	24 (28)	30 (41)	0.510
ALT*^,a,d ^(U/L)	21 (21.5)	29 (65)	0.750
Albumin*^,a,d^ (g/L)	2.2 (0.45)	2.4 (0.9)	0.944
Hemoglobin*^,a,d^ (g/dl)	8.8 (2.8)	8.7 (2.3)	0.954
Lactate*^,a,d^ (mmol/L)	2.2 (1.35)	1.8 (1.2)	0.184

Renal damage developed to varying degrees in 21 of the patients, defined as the nephrotoxicity (NT) group, while 39 patients showed no signs of renal damage, defined as the non-nephrotoxicity (non-NT) group. The median age of the NT group was 72 years (17), while that of the non-NT group was 52 years (46) (p=0.008). The median APACHE-2 score of the NT group was 14 (17), while that of the non-NT group was 21 (16); they were statistically similar (p=0.055). *Acinetobacter baumannii* was the most commonly isolated microorganism in the NT group (57.1%), and *Klebsiella pneumonia* was the second most commonly isolated microorganism (28.6%). In the non-NT group, the same microorganisms were detected at rates of 46% and 30%, respectively (p=0.644). The most commonly isolated site of microorganisms was the lungs in both the NT and non-NT groups, with rates of 85.7% and 69.2%, respectively.

The median basal GFR values of the patients were found to be 57.23 (39.82) in the NT group and 139.23 (168.62) in the non-NT group (p=0.01). In terms of mortality rates, both groups were similar. The mortality rates of the NT and non-NT groups were 14/21 (66.7%) and 20/39 (51.3%), respectively (p=0.251). There was no statistically significant difference between the infection marker (procalcitonin) and albumin values of the two groups (Table 3). None of our patients required renal replacement therapy, and none developed end-stage renal failure. We found that the duration of polymyxin B use, BMI, and advanced age were independent risk factors for this rate (Table 4).

**Table 3:** Nephrotoxicity predictors a: number and %, * median (interquartile range), c: chi-squared test, d: patients had use of multiple nephrotoxic agents, KDIGO: Kidney Disease Improving Global Outcomes, NT: nephrotoxicity, NSAIDs: non-steroidal anti-inflammatory drugs, ACEI: angiotensin-converting-enzyme inhibitors

	NT group	Non-NT group	p-value
KDIGO^a,c^	21 (35%)	39 (65%)	
Stage 1	12 (57,1%)	-	
Stage 2	6 (28,6%)	-	
Stage 3	3 (14,3%)	-	
Source of infection^a,c^			0,3489
Pulmonary	18 (85,7%)	27 (69,2%)	
Cerebrovascular	-	2 (5,1%)	
Genitourinary	-	3 (7,7%)	
Intra-abdominal	3 (14,3%)	7 (17,9%)	
Reason for hospitalization^a,c^			0,578
Neurological disease	-	1 (2,6%)	
Pneumonia	17 (81%)	27 (69,2%)	
Sepsis	4 (19%)	9 (23,1%)	
Cardiovascular disease	-	2 (5,1%)	
Concomitant nefrotoxins^ a,c,d^			0,920
Carbapenem	12 (57,1%)	26 (66,7%)	
Aminoglicoside	5 (23,8%)	5 (12,8%)	
Amphotericin b	1 (4,8%)	-	
Vancomycin	1 (4,8%)	3 (7,7%)	
Tigecycline	3 (14,2%)	9 (23,1%)	
NSAIDs	1 (4,8%)	1 (2,6%)	
ACEI	2 (9,5%)	4 (10,3%)	
Norepinephrine	7 (33,3%)	19 (48,7%)	0,251
Concomitant nefrotoxins^a,c^			0,935
One nephrotoxic drug	11 (61,9%)	20 (43,6%)	
Receipt of ≥2 nephrotoxic drugs	10 (28,6%)	19 (52,8%)	
Concomitant disease^a,c^			<0,011
One concomitant disease	8 (38,1%)	24 (61,5%)	
Receipt of ≥2 concomitant disease	12 (57,1%)	9 (23,1%)	
Intensive care stay*^,b,c^	15 (17)	18 (8)	0,294
Polymyxin B day of use*^,b,c^	14 (13)	8 (7)	0,006
Exitus^a,c^	14 (66,7%)	20 (51,3%)	0,251

**Table 4:** Multivariable logistic regression analysis GFR: glomerular filtration rate

	Beta	Wald	p	Odds ratio	Odds ratio 95% safety margin
BMI	-0.254	3.955	0.047	0.776	0.604-0.996
Age	-0.054	3.955	0.047	0.947	0.898-0.999
Concomitant disease		2.993	0.224		
Concomitant disease (only one)	0.533	0.131	0.717	1.704	0.095-30.565
Concomitant disease (two and more)	1.476	2.959	0.085	4.375	0.814-23.507
Creatinine	0.155	0.089	0.766	1.167	0.421-3.236
GFR	0.012	1.604	0.205	1.012	0.994-1.031
Polymyxin B treatment duration	-0.169	4.934	0.026	0.845	0.728-0.980

## Discussion

In patients treated with polymyxin B, the rate of renal injury was 35%. We found that the duration of polymyxin B use, BMI, and advanced age were independent risk factors for this rate. None of the patients in our study needed renal replacement therapy, and none progressed to end-stage renal failure. With the cessation of treatment, renal damage regressed in all patients who developed AKI.

Nephrotoxicity caused by polymyxin B use has been found in different studies [5,6] within a wide range, specifically 4% to 60%. Such a wide range is understandable because these studies were conducted in different patient populations and ethnic groups and with different nephrotoxicity criteria. Our result of 35% nephrotoxicity is not surprising in this regard. The usual suspects for the predictors of nephrotoxicity are the total high dose of polymyxin B, use of a loading dose, hypoalbuminemia, advanced age, high baseline creatinine, and use of additional nephrotoxic agents. 

Chang et al. [8] conducted a multicenter study in China examining polymyxin B nephrotoxicity and its predictors and found that the use of two or more nephrotoxic agents was an independent risk factor for polymyxin B nephrotoxicity. In a study by Peagau et al. [9] in 2011, the use of three or more nephrotoxic agents increased polymyxin nephrotoxicity. This finding is hardly surprising considering that the combination of drugs with a harmful effect on nephrons can increase nephrotoxicity due to the increasing burden on them. Therefore, in patients treated with polymyxin group antibiotics, we advise caution when using additional nephrotoxic agents and to monitor closely for possible nephrological damage. In addition, in previous studies [10-13] with polymyxins, various comorbidities have been found to be effective on renal damage. Although we did not find any difference caused by single comorbidity on nephrotoxicity, we observed in the univariate analysis that there was significantly more nephrotoxicity in patients with two or more comorbidities; however, we could not confirm this data in the multivariate analysis (Table 4). Regarding additional nephrotoxic agents, we could not show any statistical significance, but we believe that this is due to the study’s insufficient sample size.

Findings on the loading and total daily dose of polymyxin B are rather controversial. It has been shown that a stable serum concentration can be achieved swiftly with a loading dose of 2-2.5 mg/kg. Some studies suggest that the loading dose is one of the main factors for renal damage [8], and, in a similar study [14] that subjected colistin, the loading dose was reported to be a cause of the significant difference in renal damage, with 77.3% compared to 23.7%. However, in a study by Nelson et al. [15], no significant difference in AKI was found between patients treated with polymyxin B with or without a loading dose (p=0.3), and it was concluded that the risk of AKI is not associated with the use of loading dose. Since our treatment protocol included a loading dose of polymyxin B for every patient in our care, it is impossible to differentiate the effect of the loading dose. However, with our findings, we can argue that the dose and duration of the drug has an effect on the nephrotoxicity of polymyxin B because both were higher in patients who developed nephrotoxicity.

The univariate analysis showed significantly lower baseline GFR and higher creatinine values in the NT group, while the multivariate analysis did not show a similar result. In a study by Mendes et al. [16], a higher incidence of nephrotoxicity was observed in patients with high baseline creatinine levels, and, similarly, in a case series by Pastewski et al. [17], the effect of the baseline creatinine value was found to be related to AKI. In a study by Han et al. [18], a close relationship was found between the baseline serum creatinine level and polymyxin B toxicity in 54 patients. However, there are also many studies in which the effect of baseline creatinine values is found to be insignificant [19,20]. We believe that low GFR values may be associated with nephrotoxicity caused by not only polymyxins but all nephrotoxic agents, and we interpret this as the greater exposure of the already decreased renal reserve to toxic agents. However, we could not demonstrate this in our study.

In our study, no significant difference was found in terms of mortality between the NT and non-NT groups, which is inconsistent with previous studies [21-23], which show higher mortality in patients with developed nephrotoxicity. We believe that this contradicting result may be due to the small number of patients in our study.

In a study conducted by Kubin et al. [5], it was found that a BMI higher than 25 kg/m2 is a risk factor for polymyxin B-associated AKI. Similarly, in our study, a significant relationship between high BMI and AKI was detected. This result may be due to the tendency of clinicians to use higher doses based on the patient’s actual body weight, rather than the ideal body weight recommended for the drug (which could be the result of concerns about multidrug-resistant gram-negative bacteria and clinical/institutional strategies). In addition, confusion still persists about how to dose obese and non-obese people and those with and without renal failure with these drugs.

Advanced age was found to be an independent risk factor for polymyxin B nephrotoxicity. Previous studies [10,12,22,24-27] on polymyxins and their nephrotoxic effects have shown a relationship between advanced age and polymyxin nephrotoxicity. We attribute this relationship to the age-related decrease in renal reserves, which results in increased sensitivity to nephrotoxic agents. However, it is unknown whether there is a linear relationship between age and nephrotoxicity caused by polymyxins [28].

Our study has several limitations. First, it is a retrospective study. In addition, most of our patients were septic and in second- and third-level ICUs, which is a condition in itself known to have serious effects on renal function. Polymyxin B was often combined with other antibiotics in our patients and, in our study group, was most commonly combined with carbapenems, making it difficult to create a control group to observe nephrotoxicity following polymyxin B use. Our study is also a single-center study with a single dosage regimen, which ensures a homogenous distribution of the groups; however, unfortunately, this situation does not allow for comparison with other dosing strategies. Finally, since our study is retrospective, many factors such as the rate of drug administration, fluid balance at the time of administration, and the timing of administration of other nephrotoxic agents could not be evaluated.

## Conclusions

It is evident that polymyxin B causes renal damage in ICU patients but to a lesser degree than colistin. We found that advanced age, higher BMI, and longer duration of polymyxin B use increase the risk of nephrotoxicity. Therefore, we recommend close monitoring of renal function and prompt intervention, particularly in patients with risk factors, during polymyxin B use. In addition, we believe that randomized controlled studies need to be conducted to gain more knowledge about the nephrotoxicity of polymyxin B. In order to avoid long-term use in patients using polymyxin b, we would like to emphasize the importance of frequent control of indications and necessity, and the importance of dose adjustment in elderly and patients with high bmi.

## References

[1] Li J, Nation RL, Turnidge JD, Milne RW, Coulthard K, Rayner CR, Paterson DL (2006). Colistin: the re-emerging antibiotic for multidrug-resistant Gram-negative bacterial infections. Lancet Infect Dis.

[2] Zavascki AP, Goldani LZ, Li J, Nation RL (2007). Polymyxin B for the treatment of multidrug-resistant pathogens: a critical review. J Antimicrob Chemother.

[3] Omrani AS, Alfahad WA, Shoukri MM, Baadani AM, Aldalbahi S, Almitwazi AA, Albarrak AM (2015). High dose intravenous colistin methanesulfonate therapy is associated with high rates of nephrotoxicity; a prospective cohort study from Saudi Arabia. Ann Clin Microbiol Antimicrob.

[4] Markou N, Apostolakos H, Koumoudiou C, Athanasiou M, Koutsoukou A, Alamanos I, Gregorakos L (2003). Intravenous colistin in the treatment of sepsis from multiresistant gram-negative bacilli in critically ill patients. Crit Care.

[5] Kubin CJ, Ellman TM, Phadke V, Haynes LJ, Calfee DP, Yin MT (2012). Incidence and predictors of acute kidney injury associated with intravenous polymyxin B therapy. J Infect.

[6] Ramasubban S, Majumdar A, Das PS (2008). Safety and efficacy of polymyxin B in multidrug resistant gram-negative severe sepsis and septic shock. Indian J Crit Care Med.

[7] Kidney Disease: Improving Global Outcomes (KDIGO) Acute Kidney Injury Work Group (2012). KDIGO Clinical Practice Guideline for Acute Kidney Injury. Kidney Inter Suppl.

[8] Chang K, Wang H, Zhao J (2022). Risk factors for polymyxin B-associated acute kidney injury. Int J Infect Dis.

[9] Pogue JM, Lee J, Marchaim D (2011). Incidence of and risk factors for colistin-associated nephrotoxicity in a large academic health system. Clin Infect Dis.

[10] Akajagbor DS, Wilson SL, Shere-Wolfe KD, Dakum P, Charurat ME, Gilliam BL (2013). Higher incidence of acute kidney injury with intravenous colistimethate sodium compared with polymyxin B in critically ill patients at a tertiary care medical center. Clin Infect Dis.

[11] Crass RL, Rutter WC, Burgess DR, Martin CA, Burgess DS (2017). Nephrotoxicity in patients with or without cystic fibrosis treated with polymyxin B compared to colistin. Antimicrob Agents Chemother.

[12] Gauthier TP, Wolowich WR, Reddy A, Cano E, Abbo L, Smith LB (2012). Incidence and predictors of nephrotoxicity associated with intravenous colistin in overweight and obese patients. Antimicrob Agents Chemother.

[13] Min KL, Son ES, Kim JS, Kim SH, Jung SM, Chang MJ (2018). Risk factors of colistin safety according to administration routes: intravenous and aerosolized colistin. PLoS One.

[14] Rigatto MH, Oliveira MS, Perdigão-Neto LV (2016). Multicenter prospective cohort study of renal failure in patients treated with colistin versus polymyxin B. Antimicrob Agents Chemother.

[15] Nelson BC, Eiras DP, Gomez-Simmonds A (2015). Clinical outcomes associated with polymyxin B dose in patients with bloodstream infections due to carbapenem-resistant gram-negative rods. Antimicrob Agents Chemother.

[16] Mendes CA, Cordeiro JA, Burdmann EA (2009). Prevalence and risk factors for acute kidney injury associated with parenteral polymyxin B use. Ann Pharmacother.

[17] Pastewski AA, Caruso P, Parris AR (2008). Parenteral polymyxin B use in patients with multidrug-resistant gram-negative bacteremia and urinary tract infections: a retrospective case series. Ann Pharmacother.

[18] Han L, Xu FM, Zhang XS (2022). Trough polymyxin B plasma concentration is an independent risk factor for its nephrotoxicity. Br J Clin Pharmacol.

[19] Holloway KP, Rouphael NG, Wells JB, King MD, Blumberg HM (2006). Polymyxin B and doxycycline use in patients with multidrug-resistant Acinetobacter baumannii infections in the intensive care unit. Ann Pharmacother.

[20] Ouderkirk JP, Nord JA, Turett GS, Kislak JW (2003). Polymyxin B nephrotoxicity and efficacy against nosocomial infections caused by multiresistant gram-negative bacteria. Antimicrob Agents Chemother.

[21] Elias LS, Konzen D, Krebs JM, Zavascki AP (2010). The impact of polymyxin B dosage on in-hospital mortality of patients treated with this antibiotic. J Antimicrob Chemother.

[22] Rigatto MH, Behle TF, Falci DR (2015). Risk factors for acute kidney injury (AKI) in patients treated with polymyxin B and influence of AKI on mortality: a multicentre prospective cohort study. J Antimicrob Chemother.

[23] Tuon FF, Rigatto MH, Lopes CK, Kamei LK, Rocha JL, Zavascki AP (2014). Risk factors for acute kidney injury in patients treated with polymyxin B or colistin methanesulfonate sodium. Int J Antimicrob Agents.

[24] Balkan II, Dogan M, Durdu B (2014). Colistin nephrotoxicity increases with age. Scand J Infect Dis.

[25] Katip W, Uitrakul S, Oberdorfer P (2017). Clinical outcomes and nephrotoxicity of colistin loading dose for treatment of extensively drug-resistant Acinetobacter baumannii in cancer patients. Infect Drug Resist.

[26] Phe K, Lee Y, McDaneld PM (2014). In vitro assessment and multicenter cohort study of comparative nephrotoxicity rates associated with colistimethate versus polymyxin B therapy. Antimicrob Agents Chemother.

[27] Temocin F, Erdinc S, Tulek N, Demirelli M, Bulut C, Ertem G (2015). Incidence and risk factors for colistin-associated nephrotoxicity. Jpn J Infect Dis.

[28] Nation RL, Rigatto MH, Falci DR, Zavascki AP (2019). Polymyxin acute kidney injury: dosing and other strategies to reduce toxicity. Antibiotics (Basel).

